# Exploring the Use of Dimethyl Fumarate as Microglia Modulator for Neurodegenerative Diseases Treatment

**DOI:** 10.3390/antiox9080700

**Published:** 2020-08-03

**Authors:** Maria Rosito, Claudia Testi, Giacomo Parisi, Barbara Cortese, Paola Baiocco, Silvia Di Angelantonio

**Affiliations:** 1 Center for Life Nanoscience, Istituto Italiano di Tecnologia, 00161 Rome, Italy; maria.rosito@iit.it (M.R.); claudia.testi@iit.it (C.T.); giacomo.parisi@iit.it (G.P.); 2 Nanotechnology Institute, CNR-Nanotechnology Institute, Sapienza University, 00185 Rome, Italy; barbara.cortese@nanotec.cnr.it; 3 Department of Biochemical Sciences “A. Rossi Fanelli” Sapienza University, 00185 Rome, Italy; 4 Department of Physiology and Pharmacology, Sapienza University, 00185 Rome, Italy

**Keywords:** dimethyl fumarate, microglia, neurodegeneration, neuroinflammation, iron metabolism, alternative compounds, antioxidants, ferritin, brain

## Abstract

The maintenance of redox homeostasis in the brain is critical for the prevention of the development of neurodegenerative diseases. Drugs acting on brain redox balance can be promising for the treatment of neurodegeneration. For more than four decades, dimethyl fumarate (DMF) and other derivatives of fumaric acid ester compounds have been shown to mitigate a number of pathological mechanisms associated with psoriasis and relapsing forms of multiple sclerosis (MS). Recently, DMF has been shown to exert a neuroprotective effect on the central nervous system (CNS), possibly through the modulation of microglia detrimental actions, observed also in multiple brain injuries. In addition to the hypothesis that DMF is linked to the activation of NRF2 and NF-kB transcription factors, the neuroprotective action of DMF may be mediated by the activation of the glutathione (GSH) antioxidant pathway and the regulation of brain iron homeostasis. This review will focus on the role of DMF as an antioxidant modulator in microglia processes and on its mechanisms of action in the modulation of different pathways to attenuate neurodegenerative disease progression.

## 1. Introduction

An increasing number of reports have highlighted the importance of dimethyl fumarate (DMF) as a key active compound, employed in a plethora of therapeutic applications. For more than four decades, DMF and other derivatives of fumaric acid ester compounds have been used in the treatment of psoriasis [1,2]. Only in 2013 the oral formulation of this compound was approved for the treatment of adults with relapsing forms of multiple sclerosis (MS) [3,4] and currently, it is the most successful chemical compound (i.e., Tecfidera, Biogen) for relapsing-remitting MS [5]. MS is the most common inflammatory disorder of the central nervous system (CNS) in young adults between 20 and 40 years of age. It is considered a prototypic organ-specific autoimmune disease, targeting the CNS with inflammatory lesions, demyelination, axonal/neuronal damage, and metabolic changes [6,7]. In particular, many studies on the immune system of MS patients indicated that T and B cells, and probably also autoantibodies are key factors contributing to its immunopathogenesis [6,8,9,10]. Indeed, autoreactive CD4+ T cells with Th1 (secreting IFN-γ) or Th1* (secreting IFN-γ and IL-17), or those secreting IFN-γ and GM-CSF [6,11,12], play an important role in MS. On this regard, DMF immunomodulatory effects for MS treatment are achieved by the Th1 to Th2 shift and by the modulation of the dendritic cells’ function [13]. In vitro and in vivo studies showed that both DMF and its metabolite monomethyl fumarate (MMF) reduce the relapse rate and the number of new lesions in MS. In addition to the recognized effects on the immune system, DMF, which is a very reactive molecule and a natural antioxidant, exerts its modulatory actions in brain parenchyma via multiple mechanisms, acting on microglia and possibly on other brain cells. Indeed, alongside the approved therapeutic applications on MS, novel studies are exploring the possible use of DMF in the treatment of tauopathies and other neurological diseases [14].

In this review, we report the mechanisms of action of DMF as a modulator of the microglia oxidative response, shedding new light on its biochemical targets, on its beneficial properties for neurodegenerative diseases treatment and, finally, on its correlation with iron metabolism.

## 2. Biochemical Targets of DMF

**Figure 1:**
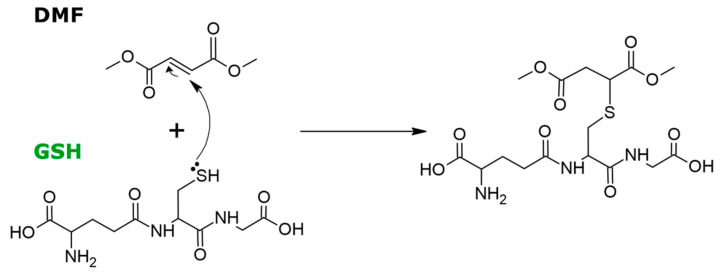
Mechanism of Michael addition, which consists in the nucleophilic addition of the cysteine thiol group of glutathione (GSH) to dimethyl fumarate (DMF) that results in a stable adduct at physiologic pH. Similarly, fumarate causes a covalent chemical modification of cysteine thiol residues of proteins, in a process known as protein succination.

DMF belongs to the family of fumaric acid esters, which are normally metabolized by intestinal esterases prior to reaching blood circulation [15,16,17,18]. As an α,β-unsaturated compound, DMF can react by a Michael addition where the nucleophilic sulfhydryl group of glutathione (γ-glutamylcysteine glycine, GSH) reacts with fumarate leading to S-(2-succino)cysteine, in a process known as protein succination (Figure 1) [19,20]. The ability of DMF to modify GSH availability, activates several cellular responses to oxidative stress including the enhancement of GSH recycling. As recently reported, DMF can also react with thiol groups of cysteine residues on a wide range of intracellular proteins, and with at least 24 proteins in neurons and astrocytes [21]. This recent study also suggests that DMF treatment may directly contribute to axonal preservation and remyelination, by modifying regulatory thiols on proteins such as cofilin-1, tubulin, and collapsin response mediator protein 2 (CRMP2). This direct protein regulation represents an alternative mechanism of action which differs from the known immunomodulatory effect of DMF in MS [21,22].

Moreover, microglia cells contain higher concentrations of GSH that can regulate important activities of enzymes such as superoxide dismutase, catalase, glutathione peroxidase, and glutathione reductase, as well as NADPH-regenerating enzymes, essential for the protection against oxidative damage.

Although the mode of action of DMF has been widely studied, the molecular basis for the many effects of DMF and MMF are still not completely elucidated, because of their multiple actions on different pathways which lead to a wide range of immunomodulatory, cytoprotective, and neuroprotective effects. Some of these are the nuclear factor erythroid-derived 2 (NRF2) antioxidant pathway, the nuclear factor kappa-light-chain-enhancer of activated B cells (NF-κB) pathway, the GSH modulation, and the hydroxycarboxylic acid receptor (HCAR2) activation.

### 2.1. DMF Activates the NRF2 Pathway via Multiple Mechanisms

To date, one of the most characterized mechanisms of action of DMF is the modulation of the NRF2 dependent pathway. Although NRF2 was first described as the master regulator of redox homeostasis, it is also known to induce the expression of about 1% of human genes which contain, in their promoter regulatory regions, an enhancer sequence termed “antioxidant response element” [23]. These genes encode a large variety of cytoprotective proteins implicated in biotransformation, antioxidant reactions and inflammation, by modifying metabolic programs [24]. Since oxidative stress is implicated as a contributing factor to the pathology of numerous neurodegenerative disorders, at a tissue and cellular level, the enhancement of these NRF2 targeted genes by treatment with DMF and MMF, protects the CNS against oxidative insults in neurological diseases and reduces proinflammatory responses [25,26]. Specifically, upon translocation into the nucleus, NRF2 enhances the transcription of a subset of genes involved in detoxification and antioxidant responses, including heme oxygenase-1 (HO-1), NAD(P)H quinoline oxidoreductase, glutathione S-transferase superoxide dismutase-2 (SOD2), sulfiredoxin-1, ferritin heavy chain 1 [27,28], and other antioxidant proteins. Prior to its nuclear translocation, NRF2 is retained in the cytosol by the interaction with Kelch-like ECH-associated protein (KEAP1), which indirectly mediates the proteasomal degradation of NRF2 [29,30]. The oxidation of cysteines in KEAP1 relieves binding and allows NRF2 to accumulate, enter the nucleus, and exert its activity [29,31,32].

So far, NRF2 activation has been achieved with satisfactory pharmacokinetic and pharmacodynamic properties using sulforaphane, CDDO-methyl ester (also known as bardoxolone methyl), and DMF [33]. Among all, the most effective has been DMF due to the dissociation of KEAP1-NRF2 complex, stable in the cytoplasm under homeostatic conditions. Although the exact molecular mechanism is not completely understood, due to the lack of knowledge of the structural determinants of the KEAP1-NRF2 complex [34], it is generally accepted that DMF preferentially oxidizes the sulfhydryl (-SH) group on Cys151. Upon a conformational change, NRF2 dissociates from the complex and migrates to the nucleus [35]. DMF has been reported to be more efficient in inducing this nuclear translocation compared to other esters of fumarate (i.e., monomethyl fumarate) [36].

The NRF2 nuclear localization is generally associated with a subsequent increase in GSH synthesis and recycling in neuronal cells. As insufficient NRF2 signaling during chronic oxidative stress is associated with several diseases including neurodegenerative, vascular, and metabolic disorders as well as cancer, the DMF-mediated modulation of the KEAP1-NRF2 complex is becoming increasingly explored as a potential therapeutic strategy.

It has been recently highlighted that NRF2 can be modulated by DMF also through a KEAP1 independent pathway. Specifically, Cuadrado and colleagues demonstrated that DMF, through the activation of the PI3K/AKT pathway, promotes NRF2 activation, counteracting inflammation and neurodegeneration [14]. This effect is mediated by the phosphorylation and thus the inhibition of glycogen synthase kinase-3β (GSK-3β). Indeed, among the intracellular actions of GSK-3β, the phosphorylation of critical residues of NRF2 represents an important pathway for ubiquitin-proteasome degradation, leading to increased inflammation [37].

For this reason, GSK-3β inhibition has become a promising approach in the treatment of different diseases associated to its over expression [38,39]. Indeed, changes in GSK-3β activity have been shown to regulate intracellular substrates involved in neuronal polarization, including collapsin response mediator protein 2 (CRMP2) and TAU, active players of microtubule dynamics.

**Figure 2:**
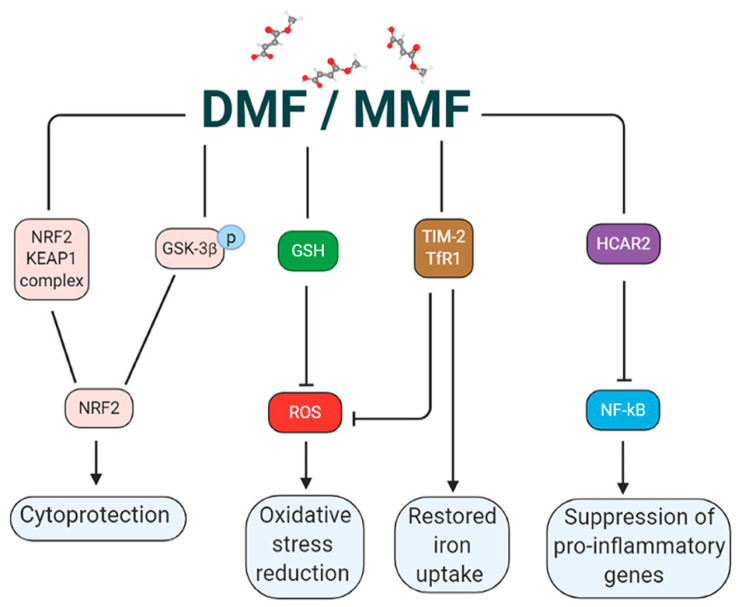
Molecular pathways and relative biochemical targets modulated by DMF (Figure created in BioRender.com).

Thus, DMF activation of NRF2 through two distinct proteolytic pathways, a KEAP1 dependent one and the other mediated by GSK-3β phosphorylation (Figure 2), makes this compound a promising therapy for neurodegenerative diseases as tauopathies, where either KEAP1 or GSK-3β activities are altered [14].

### 2.2. DMF Modulates GSH

For self-protection against ROS-mediated damage, eukaryotic cells are equipped with an efficient antioxidative defense mechanism, based on GSH, glutathione peroxidase (GPx), and superoxide dismutase (SOD): these are endogenous antioxidants, which scavenge the free radicals and protect the cells against oxidative stress. It has been reported that DMF exerts its antioxidant effect (Figure 2) also through a delicate control of the GSH levels. Indeed, the GSH concentration, which is typically in the millimolar range [40], is essentially obtained by a tuned modulation between the GSH depletion through protein succinylation, and the activation of a cascade of antioxidative enzymes such as superoxide dismutase, glutathione peroxidase, and glutathione reductase, as well as NADPH-regenerating enzymes, which promote GSH formation [13,41,42,43,44]. An intriguing hypothesis is that the DMF interaction with GSH leads to the inhibition of NF-κB nuclear translocation and transcriptional activity, which is a critical step in the regulation of inflammation [45,46].

### 2.3. DMF Activates HCAR2 in a NRF2 Independent Pathway

**Figure 3:**
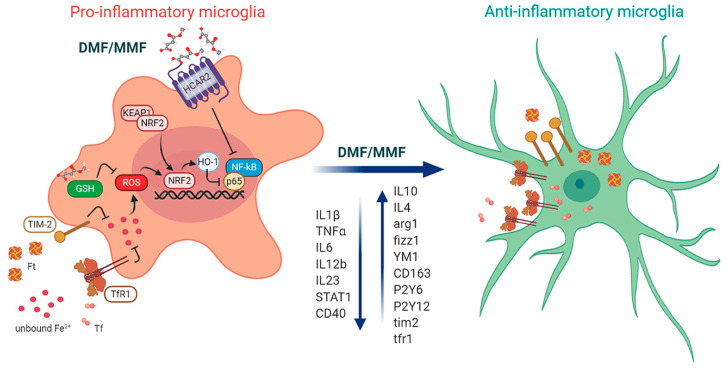
Effects of monomethyl fumarate (MMF)/DMF treatment on microglia and related molecular pathways involved in reducing inflammation. **Left:** In pro-inflammatory microglia, DMF/MMF: (*i*) activate NRF2 pathway in a KEAP1-dependent manner; (*ii*) inhibit NF-κB pathway via HCAR2 receptor binding; (*iii*) modulate GSH levels, thus depleting ROS and activating the NRF2 pathway in a KEAP1-independent manner; (*iv*) upregulate TIM-2 and TfR1 (in mice, receptors responsible for Ft and Tf uptake respectively), thus reducing the intracellular levels of unbound Fe(II) and of ROS. **Centre and right:** These effects are compatible with a shift towards the anti-inflammatory phenotype, by downregulating pro-inflammatory genes and upregulating the anti-inflammatory ones. TIM-2 and TfR1 are over-expressed, thus allowing the uptake of Ft and Tf-bound iron inside the cells. ***Legend***: DMF = dimethyl fumarate, MMF = monomethyl fumarate, NRF2 = nuclear factor (erythroid-derived 2)-like 2, NF-κB = nuclear factor–κB, GSH = glutathione, HO-1 = hemoxygenase-1, ROS = reactive oxide species, TIM-2 = T-cell immunoglobulin mucin domain 2 protein, TfR1 = transferrin receptor 1, Tf = transferrin, Ft = ferritin (Figure created in BioRender.com).

Besides being a prominent activator of NRF2, numerous studies showed that DMF acts also as a strong direct agonist of HCAR2, a G-protein coupled membrane receptor expressed in immune cells such as neutrophils, dendritic cells, macrophages, and microglia, whose activation induces a robust anti-inflammatory signaling. DMF effects on HCAR2 receptor, independently from NRF2, have been shown to be neuroprotective, reducing spinal cord infiltration of blood-borne neutrophils [47]. In addition, using the same HCAR2 mediated pathway, MMF activates the CaMKK2-AMPK-SIRT1 axis in microglia, reducing the expression of inflammatory genes [22]. Despite the evidence reported on brain immune cells, the functional expression of HCAR2 on neurons remains unclear, as the level of HCAR2 mRNA in murine and human neuronal cell lines is lower, compared to primary mouse microglia and human naïve blood monocytes [48]. This suggests that potential neuroprotective strategies triggered by DMF or MMF are mostly mediated by microglial activation (Figure 3).

## 3. DMF Modulates Microglia Functions under Homeostatic and Pathological Conditions

Besides the well-described actions on the immune system, DMF treatment directly modulates CNS functions, affecting microglia. In healthy adult tissue, microglia behave as an extremely plastic and pleiotropic cell type. They are active sentinels involved in the maintenance of a homeostatic CNS microenvironment [49]: in response to insults or injuries, microglia acquire diverse and complex phenotypes, that can exacerbate the cytotoxic response or promote immunoresolution and tissue remodeling [50,51,52,53]. Under in vivo pathological conditions, the phenotype of individual microglia resides along the continuum between cytotoxic/pro-inflammatory and alternatively/anti-inflammatory activated microglia, which is reflected in the unique expression signature of cell-surface markers [54]. In order to counteract neurodegenerative pathologies, one of the most challenging approaches is to therapeutically target microglia in order to redirect this cell type from detrimental to beneficial function. Microglia are the primary source of proinflammatory and anti-inflammatory cytokines, active players in the modulation of neuroinflammation and mediators of a broad spectrum of cellular responses [52,55]. Indeed, microglia activation, irrespective of the particular polarized states, is a salient feature of neuro-inflammation that is prominent in almost all neurodegenerative diseases such as Alzheimer’s disease (AD), Parkinson’s disease (PD), amyotrophic lateral sclerosis (ALS), and MS. The neuroprotective effects of DMF in neuroinflammation have been massively highlighted during the last decade and a number of research groups have focused on fumaric acid esters antioxidant activated pathways and on their ability in modulating microglia functions via activation of NRF2, promoting its antioxidant abilities [22,56,57]. The antioxidant action of DMF on microglia, mediated by NRF2 activation, promotes the reduction of neuroinflammation by lowering the expression of IL1β, TNFα, IL12b, IL23, STAT1, and CD40 [25,26], (Figure 3). In addition to the well accepted mechanism involving DMF on NRF2 in a KEAP1-dependent manner [33,58], Cuadrado and colleagues proposed an alternative KEAP1 independent mechanism, that controls NRF2 protein stability, involving the PI3K-AKT-GSK-3β axis in microglia mediated neuroprotection in mouse embryonic fibroblasts [14]. This was also demonstrated in a tauopathy model of chronic neuroinflammation, where DMF treatment reduces astrogliosis and microgliosis triggered by TAU P301L expression, decreasing the level of pro-inflammatory markers such as IL1β and inducible nitric oxide synthase (iNOS), and promoting the shift of microglia towards a ramified resting shape. The KO of NRF2 prevented the beneficial effect of DMF on astrocytes and microglia, thus corroborating the hypothesis that DMF anti-inflammatory effects are at least in part mediated by glial NRF2 activation.

In addition, DMF has been shown to exert its immunomodulatory effect in several NRF2-independent mechanisms within microglia. To achieve its therapeutic effects in the experimental autoimmune encephalomyelitis (EAE) mouse model of MS, the DMF (and MMF) mediated activation of HCAR2 is required not only to inhibit neutrophil infiltration [47], but also to modulate microglia phenotype [22]. Indeed, microglia express HCAR2, and the binding of MMF to HCAR2 can induce CaMKK activation, thus triggering the AMPK/SIRT1 axis, which strongly contribute to mitigate inflammation through the inhibition of the NF-κB pathway and subsequent pro-inflammatory cytokine production [22]. Moreover, in acute corticostriatal slices of EAE mice, DMF application reduces glutamatergic transmission acting at the presynaptic level by modulating glutamate release. This effect was mimicked by microglia conditioned medium, when pro-inflammatory polarized microglia were treated with MMF, suggesting an indirect, microglia mediated, neuroprotective effect at a synaptic level. Thus, through a novel microglia HCAR2-dependent pathway, DMF reduces neuroinflammation and restores synaptic alterations in the EAE mouse model of MS [22].

On the other hand, GSH is a potent effector of DMF also in microglia. Microglia, which in neuropathological conditions are frequently exposed to high ROS levels, display a robust antioxidant defense system against oxidative damage. GSH levels, which are particularly high in homeostatic microglia [41,42,43], decrease with age, thus revealing an age-dependent increase in the vulnerability of microglia to oxidative stress [59]. Antioxidant mechanisms mediated by DMF through the modulation of glial GSH are not completely understood, as the ROS balance is influenced by the complex interactions of multiple biochemical pathways. In physiological conditions, GSH scavenges superoxide anions and other ROS, either directly by coupling to oxidation to GSSG, or via enzyme-catalyzed reactions. Such reactions include the GPx-catalyzed oxidation of GSH and glutaredoxin (GRx)-mediated reduction of oxidized cysteine residues in proteins. Microglia display the highest activity of GPx amongst brain cells in both rats [60] and humans [61], and the expression of the enzyme increases directly in response to oxidative stress or excitotoxin-mediated cell damage [62]. DMF reacts spontaneously with the thiol group of GSH, activating the NRF2 pathway by depleting cellular levels of GSH and reducing cell viability in a dose-dependent manner (Figure 2) [63]. In addition, astrocytes, essential for the maintenance of the global redox balance in the CNS under normal [64] or pathological conditions [65], express many antioxidant proteins at high levels. Glial oxidant production and associated antioxidant functions are physiologically relevant processes necessary to maintain and support the neuronal circuitry and the brain parenchyma homeostasis; in pathological conditions, they participate to the oxidative damage and the imbalance of autocrine and paracrine redox signaling. Overexpression of NRF2 in astrocytes is sufficient to mediate neuroprotection in animal models of PD. This effect may arise from the export of GSH from astrocytes [66] or alternatively, from the astrocytic ROS uptake.

Wilms and colleagues demonstrated that induction of NRF2 in astrocytes increases production of GSH, which can exert cytoprotective effects for glial cells and neighboring neurons. They suggested also that DMF treatment on rat astrocytes is able to reduce cytokine (IL1α, IL6, and TNFα) mRNA levels, although levels of NOS2 mRNA were not significantly reduced [67]. Conversely, Pars and colleagues underlined that fumaric acids do not directly influence neuroprotective factors gene expression in rodent astrocytes. Indeed, DMF and MMF have no effect on growth factors and cytokine expression in lipopolysaccharide (LPS) stimulated astrocytes. This suggests that the proposed neuroprotective effect of fumaric acid is not mediated by direct stimulation of neurotrophic factors in astrocytes but is rather mediated by other pathways or indirect mechanisms via other glial cells, like microglia [68].

Another biochemical pathway that may be involved in the microglia mediated protective action of DMF is the modulation of iron homeostasis. Indeed, while a strong correlation between free iron and cellular damage in MS and neurodegenerative disorders has been reported, with a significant accumulation of unbound iron in the brain parenchyma [69,70], microglia cells have been reported to store iron-bound ferritin and to release it to oligodendrocytes to support myelination [71,72]. Recent evidence in primary murine microglia indicated that DMF treatment both interacts tightly with iron metabolism, through the increase of ferritin-bound iron uptake, and promotes an anti-inflammatory neuroprotective microglia phenotype, likely contributing to beneficial effects on MS [73,74]. Another action of DMF on microglia activity is the downregulation of purinergic receptors P2Y12 and P2Y6 transcripts in both homeostatic and proinflammatory phenotype [74]: this has the beneficial effect to reduce the ATP-induced reactivity towards an external injury and thus to promote protective, anti-inflammatory phenotypes (Figure 3).

## 4. DMF Targets in Neurodegenerative Disorders

Neurodegenerative diseases such as AD, PD, ALS, Huntington’s disease (HD) are characterized by the failure of specific populations of neurons, such as pyramidal neurons in AD, dopaminergic neurons in PD, motor neurons in ALS, and striatal medium spiny neurons in HD. However, several recurrent features in all of these diseases include oxidative stress and glial activation, in which microglia and astrocytes acquire different phenotypes that can be either protective or detrimental to neurons [75]. In this context, the modulatory effect exerted by DMF on both oxidative stress and microglia functions can be beneficial in the cotreatment of neurodegenerative disorders. Indeed, there is a need to develop therapeutic strategies that can impede or halt the disease through the modulation of the central and peripheral immune system by controlling the existing neuroinflammation, and by reducing oxidative stress [76]. Currently, there are no proven protective treatments available for patients with neurodegenerative disorders, while some different drugs can provide relief from the associated symptoms but are less effective as the disease progresses.

DMF treatment has been shown to ameliorate disease hallmarks and symptoms in disease models (cellular and mouse models) not only in MS, but also in AD, PD, ALS, and in Friedreich’s ataxia (FA), an inherited neurodegenerative disorder resulting from decreased expression of the mitochondrial protein frataxin, for which there is no approved therapy [77].

AD is a chronic and progressive neurodegenerative disorder, which is accounted as the primary cause of dementia in the aging population [78,79]. At a cellular and molecular level, AD is characterized by extracellular amyloid-beta peptides aggregation, intracellular deposits of hyperphosphorylated TAU, neurodegeneration, and microglia and astrocyte activation in the brain and retina [80,81]. Up to now, no effective disease-modifying therapies are available for AD patients. The approved treatments are limited only to symptomatic management and consist mostly of acetyl-cholinesterase inhibitors and N-methyl-D-aspartate receptor antagonists that alleviate the cognitive and functional deficits for a limited time, while clinical trials focusing on amyloid-beta clearance have failed [82]. The alternative therapeutic approaches are based on the view of AD pathogenesis as a pathological network that involves the simultaneous modulation of several key biological targets, giving rise to a heterogeneous disease, strictly linked to inflammation and oxidative stress [83].

Thus, given the complexity of the AD pathology, the multi-target ligands, acting as master regulators of cellular defense mechanisms, may work synergistically to exert their efficacy in a holistic way. Among these multi-target ligands, DMF, which acts both on oxidative stress and immune response, represents the most pharmacologically effective molecule [84]. In AD in vitro models, treatment with DMF significantly reduced amyloid beta-induced cell death, TAU phosphorylation, and intracellular ROS production [84]. Moreover, DMF exerts beneficial effects in in vivo AD models, improving motor and cognitive symptoms, reducing inflammation in AD mouse models [85], and reducing neurodegeneration and microglia activation in the streptozotocin rat AD model [86].

Similar to AD and only second to AD in prevalence, PD is a neurodegenerative disease, characterized by motor symptoms that appear after an extensive loss of dopaminergic neurons of the substantia nigra [87]. Characteristic of the disease is the presence of α-synuclein (α-syn) protein aggregates in synapses and axons, which plays a central role in PD etiopathogenesis [88] and induces a prominent response of both innate and adaptive immunity, thus contributing to dopaminergic neurodegeneration and disease progression [89]. While currently no effective protective treatments are available for patients with PD [90], motor symptoms are routinely treated using drugs as levodopa and apomorphine, that however, become less effective as the disease progresses.

Pharmaceutical therapies that target inflammation and the immune response are promising approaches for the treatment of PD [91]. Persistent inflammatory responses, involving T cell infiltration and microglia activation, are common characteristics of human patients with PD and are involved in the degeneration of dopaminergic neurons [92,93]. Thus, also in this case, a multi target drug, as DMF, may provide beneficial effects in the disease treatment.

Indeed, in cellular and rodent models of PD, DMF has been reported to reduce the motor deficits, protecting neurons against α-syn toxicity, and decreasing microgliosis and astrogliosis [33,94,95].

Another rising application of DMF is in the treatment of ALS [96]. The currently approved treatments for this progressive and fatal neurodegenerative disorder of the human motor system include the anti-glutamatergic agents riluzole and the free radical scavenger edaravone [97], which both provide limited benefits. Like other neurodegenerative disorders, ALS is a complex disease characterized by the impairment of RNA metabolism and translation, protein quality control, and degradation, coupled to a strong presence of oxidative stress and neuroinflammation. Indeed, astrocytes, microglia, and T cells actively contribute to neurodegeneration and disease progression [98]. The effect of DMF as an immunomodulator and as a rebalancing drug of the inflammatory axis towards a neuroprotective phenotype, paved the way for the TEALS Study: a Phase2 ongoing clinical trial of DMF on ALS patients. Up to now, promising results at the primary endpoint show that DMF is effective in slowing disease progression [99].

Thus, DMF acting as an effective antioxidant and immunosuppressive drug may be easily repurposed to treat, in combination with specific disease-targeted drugs, a number of multifactorial neurodegenerative diseases (AD, PD, ALS, FA, among others). In this context, it has to be remarked that a recruiting phase 2 clinical trial is ongoing for the use of DMF (Tecfidera) for the treatment of age-related macular degeneration, which is one of the leading causes of irreversible blindness, characterized also by the presence of oxidative stress and inflammation (LADIGAGA- NCT04292080).

## 5. DMF as Regulator of Iron Metabolism for the Treatment of Neurodegenerative Diseases

Although the pathological hallmarks of neurodegenerative diseases such as PD, AD, ALS, and MS differ significantly, they all share some common factors: in addition to the already described inflammation and oxidative stress, increased iron levels in specific areas of the brain and cerebrospinal fluid [100] have been reported [72,101,102,103]. Indeed, possible dysfunctions of iron metabolism in the CNS have recently turned out to be one of the pivotal points upon which extensive research for pathological mechanisms in neurodegenerative diseases has been focused. Magnetic resonance imaging (MRI) and histological studies have shown global accumulation of iron levels in specific areas of the brain, depending on the disease [104,105,106]. It is unknown whether this increase might be the main cause that could lead to different syndromes or is the effect of a pre-existing condition.

In a healthy adult brain, the highest iron levels are found in oligodendrocytes, forming the myelin sheath: iron is in fact an essential element for myelination [107,108,109,110,111]. Even though extracellular iron accumulation in the brain is a typical age-related phenomenon [112], elevated iron levels in microglia can be considered a hallmark of pathological processes.

Indeed, in MS, iron accumulates in macrophages and microglia around the rim of lesions, the sites of inflammation, and the demyelinated plaques, highlighting signs of dystrophy through an MRI scan [72,113,114]. This phenomenon may be caused in MS by the pathological iron release from damaged oligodendrocytes in the extracellular space and the subsequent iron uptake by macrophages and activated microglia. This ultimately results in neurons and axons damage and in further myelin breakdown [103,104,115].

In PD, iron is elevated in neurons and microglia of the substantia nigra and the lateral globus pallidus [101,116,117,118,119,120], while in AD, it is mainly found in the parietal cortex, motor cortex, and hippocampus [105,121,122,123,124,125,126]. The accumulation of unbound iron induces the production of free radicals that deeply damages the proteins structure. This can lead to aggregation and accumulation of misfolded proteins as extracellular plaques and intracellular inclusion bodies. Hence, dysregulated iron levels might play an important role in the formation of Lewy bodies and amyloid plaques. Indeed, iron is found in PD in Lewy bodies [127,128,129], where the α-syn aggregation is favored by unbound Fe^2+^ and is blocked by deferoxamine, an iron chelator [101,130,131,132], or in AD, high iron levels colocalize with beta-amyloid plaques [73,133,134].

In order to pursue the missing link between neurodegenerative processes and iron accumulation in specific brain areas, extensive research has been carried out towards the causes of an improper iron metabolism in CNS.

As a matter of fact, iron possesses both toxic and beneficial properties for cellular health: a tightly controlled iron regulation is thus crucial for physiological processes. Iron is in fact a cofactor for a vast array of primary biochemical reactions (oxygen transport, energy metabolism, DNA synthesis, etc.). In particular, in the CNS, iron maintains the integrity of oligodendrocytes and facilitates their regeneration following injury. Moreover, it is a constitutive element of neurotransmitters and an essential component for myelin production. Nonetheless, unbound Fe(II) is toxic [135,136]: upon interaction with free oxygen, at neutral pH, it produces ROS by the Haber–Weiss process via a Fenton reaction [137]. Free ROS are deeply destructive for cells: because of their extreme reactivity, they may damage enzymes, membrane lipids, proteins, and nucleic acids. In the CNS, free radicals lead to neurotoxicity through oxidative stress, increased stress-associated cytokines release by microglia, glutamate toxicity, and defective DNA repair [102]. To avoid these harmful side effects, Fe(II) is always bound to a protein ligand, such as transferrin (Tf), ferritin (Ft), lactoferrin, hemosiderin, and many others. In particular, Tf’s main role is to transport iron in the blood and to deliver it to cells by coordinating two free ferric ions.

Iron uptake is linked to dietary sources and is mostly absorbed at the small intestine level by mature enterocytes. While the heme-iron is the most abundant form, its specific absorption mechanism is poorly characterized [135]. On the other hand, free iron, stabilized in soluble form by the low pH at the stomach and small intestine level, enters the circulation after crossing both the enterocyte apical membrane via iron-import protein divalent metal-ion transporter 1 and the basolateral membrane via the iron export protein ferroportin-1. Once in the bloodstream, Fe^3+^ binds to Tf and is delivered throughout the body tissues through the circulation. Tf-bound iron is then imported across the blood–brain barrier (BBB) [135,136].

On the other hand, Ft’s main physiological function is centered around intracellular iron storage: this hollow cage-like protein is a heteropolymer composed of heavy and light chains: the former, having ferrous oxidase activity, catalyzes the oxidation of Fe^2+^ to Fe^3+^, the latter promotes the formation of iron nuclei. Upon iron oxidation, Ft stores iron in its inert form inside the internal cavity, encapsulating up to 4500 Fe^3+^ atoms [138,139]. Since Ft can deliver much higher amounts of iron per mole than Tf, it has also important functions in iron trafficking.

These iron binding proteins are internalized in cells through several receptors, the most important one being transferrin receptor 1 (TfR1), one of the main regulators of iron homeostasis. TfR1 guarantees adequate iron supply to the cell in response to cellular demand by binding to plasma iron-carrier proteins as hereditary hemochromatosis factor (HFE, that blocks iron import in the cell), Tf, and Ft [140,141]. Upon binding of iron-loaded Tf to TfR1, a clathrin-dependent endocytosis mechanism initiates and iron is released inside the cell.

Adequate iron levels inside cells are also regulated by a specific class of proteins called iron response proteins (IRP). These are cytosolic proteins that increase mRNA levels of the aforementioned iron-regulatory proteins according to intracellular iron concentration. In mammalian cells, two IRPs exist, IRP1 or Aconitase 1 (ACO1) and IRP2. In case of cellular iron deprivation, IRPs bind specific iron responsive elements (IREs) in the 3′ untranslated region (UTR) of TfR1 mRNAs (with the consequent increase in their expression levels and enhanced iron uptake) and in the 5′ UTR of Ft mRNAs (resulting in reduced amounts of iron storage and export) [142]. The opposite happens when levels of intracellular iron rise: iron binds as Fe-S clusters to IRPs active centers and prevent their interaction with IREs [143]. In this condition, ACO1 catalyzes the isomerization of citrate to isocitrate in the tricarboxylic acid cycle. The physiological levels of all the above-mentioned proteins and IRPs are dysregulated in neurodegenerative diseases [72,101,102,103,121,144]. As already mentioned, in the normal adult brain, oligodendrocytes show the highest levels of iron, but they do not uptake it directly: microglial cells accumulate extracellular iron over time and feed them during myelination with massive transfers of iron-loaded ferritins [71,111,145,146]. In this context, internalization of Ft has been proved to be mediated by the receptor TIM-2 (T-cell immunoglobulin mucin domain 2 protein) [145], however this is valid only in mice, because this receptor is not expressed in humans [141,145,147]. As such, the human counterpart of this important mechanism for oligodendrocytes iron uptake from microglia might not be so straightforward. Indeed, human TfR1 binds both Tf and Ft, while murine TfR1 seems to recognize only Tf but not Ft [141,147] (Figure 3). Ft-bound iron internalization in mice and in humans’ brains might thus be quite different: proving this hypothesis would be an important validation for the usage of mice as animal models for neurodegenerative diseases, in which Ft-bound iron dysregulation plays an important role. Indeed, iron deposits in the above-mentioned areas of the brain colocalize with Ft, accumulated in macrophages and microglia [72,114].

Microglia, acting as the Ft-bound iron mediator between extracellular space and oligodendrocytes, play a key role in iron homeostasis regulation and myelin formation. It is noteworthy that understanding the intrinsic and extrinsic mechanisms that regulate microglial function in oligodendrocyte maturation and myelination is critical for the development of novel therapeutics that support repair also following perinatal ischemic insult. Indeed, despite the growing evidence for the role of microglia in the pathogenesis of perinatal brain insult, which leads to impaired myelination, there are no approved therapies aimed at modifying microglia activation in this context, and the hypothesis of MS drug repositioning to modulate the pro-inflammatory to an anti-inflammatory/pro-regenerative microglia response is taking place [148]. Recent data suggest that iron accumulation in microglia and macrophages can affect their polarization: its overload promotes the switch towards the pro-inflammatory phenotype [72,106,111,149]. This state is characterized by low levels of TfR1 [74] and high levels of unbound iron [72,73,150]. Instead, unbound iron content is low in anti-inflammatory state [151]: here, mRNA levels of TfR1 and TIM2 are high [73,74] (Figure 3).

Microglia proinflammatory phenotype, promoted by iron, leads to the release of stress-associated cytokines that further increases inflammation, resulting in a loop process: consequently, iron dysregulation is tightly linked to both oxidative stress and inflammation that characterize neurodegenerative disorders. Therapeutic agents that can remove iron from certain brain regions without causing detrimental iron deficiency could be effective in reducing diseases progression [121,152]. In the past, several iron chelators have been proved to be effective on AD or PD patients or cellular and animal models, as desferrioxamine [153] and deferoxamine [154,155,156]. However, the main difficulty in using iron chelators is constituted by the BBB, which is largely impermeable to these large molecules: only a small amount of them is able to reach the target in the CNS [121].

In this framework, recent data [74] showed that DMF might act not only as a neuroprotector, as discussed above, but also as a regulator of iron metabolism. In primary murine microglia, DMF has been shown in fact to upregulate TIM-2 expression and to promote Ft uptake. DMF treatment favors TIM-2 expression levels both in surveilling and in pro-inflammatory microglia, while it upregulates murine TfR1 transcripts only in the pro-inflammatory case, leaving it unchanged in the surveilling phenotype (Figure 3).

Hence, in microglia, DMF specifically targets the pathway for Ft-bound iron uptake and, as discussed in previous sections, also promotes a shift towards the anti-inflammatory phenotype: these two effects might be, thus, interconnected. These results support also the known benign influence of DMF-treated microglia on proliferation of oligodendrocyte precursor cells [157]: DMF would restore an impaired iron uptake in microglial cells by promoting TIM-2 expression on their surface and possibly stimulating the iron loaded Ft transfer to oligodendrocytes. The mechanism under which DMF would be able to modulate TIM-2 expression, however, still remains elusive. A possible explanation of DMF involvement in iron homeostasis might lie in its ability to covalently bind the thiol groups, responsible for iron binding, of ACO1, thus impairing its modulation activity of Ft, Tf, and rising TfR1 and TIM-2 expression levels. As such, the demonstration of DMF linking to ACO1 iron binding site would pave the way to the rational design of novel DMF-like drugs. Indeed, DMF has been shown to impact oligodendrocytes metabolism by upregulating succinate, fumarate, and malate levels, intermediates of Krebs cycle, suggesting a key role in the binding of ACO1: these effects influence mitochondrial function and protect from oxidant signaling [158].

DMF regulatory ability towards iron metabolism may be linked to its known immunomodulatory and stress protective effects in the treatment of MS [22], where it might have beneficial effects both on iron homeostasis and myelination. Reports in literature have shown that DMF can reduce amyloidopathy, microglia activation, and ROS production in in vitro and in vivo AD models, and, moreover, improve cognitive and motor symptoms [85,86,159]. It would be thus intriguing to prove whether these effects could be ascribed to the same positive effects on α-syn and beta-amyloid aggregations of other iron-chelators, as discussed above. Differently from them, indeed, DMF represents a true promising alternative as it can cross the BBB [160] and consequently it could specifically reach the specific target.

## 6. Potential DMF Alternatives: Emerging Anti-Inflammatory and Anti-Oxidant Compounds

**Figure 4:**
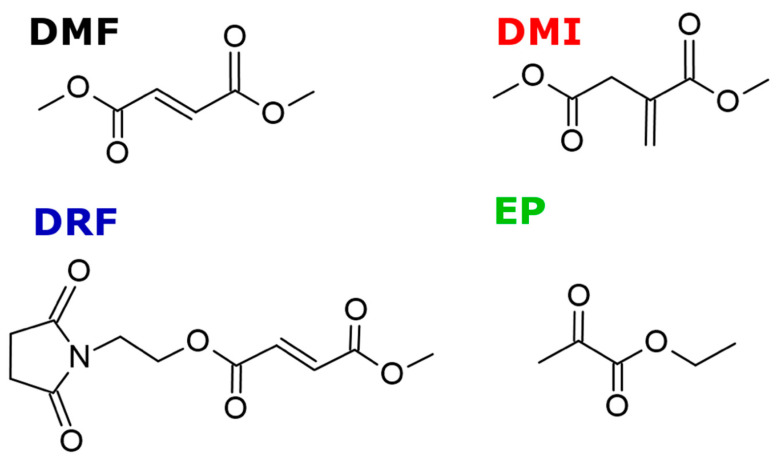
Structures of dimethyl fumarate (DMF) and some alternative compounds including dimethyl itaconate (DMI), ethyl pyruvate (EP), diroximel fumarate (DRF).

Whether considering DMF as a promising tool not only for the treatment of MS but also for neurodegenerative disorders, it is necessary to contemplate that, despite the broad efficacy, good safety, and satisfying tolerability, long term DMF administration can induce several side effects too. Indeed, DMF main metabolites, such as MMF, when further hydrolyzed to fumaric acid and recycled into the citric acid metabolism and methanol, can induce adverse gastrointestinal effects, thus requiring the research of alternative compounds with a higher tolerability (Figure 4) [17,18].

As an example, diroximel fumarate, which displays a very similar structure and chemical reactivity to DMF, has been reported to alleviate gastrointestinal pain by producing methanol as a minor metabolite [17]. Furthermore, ethyl pyruvate, a substance that is processed to pyruvate and is incorporated in the Krebs cycle comparably to fumarate, is renown to have similar MS-relevant anti-(neuro)inflammatory effects, although only if administered at higher concentrations, due to its lower efficiency in the scavenging of ROS respect to DMF [18]. Among these compounds, dimethyl itaconate (DMI), a cell-permeable itaconate derivative, has recently emerged as an alternative drug to DMF, due to its anti-inflammatory properties. Itaconate, (Figure 4), is one of the most highly induced metabolites in activated macrophages in response to inflammatory processes. It generates upon remodeling of the tricarboxylic acid cycle (TCA), an adaptation accompanying inflammatory macrophages activation, by decarboxylation of the TCA intermediate cis-aconitate. Itaconate has been shown to be produced in response to lipopolisaccaride (LPS) and certain infections by the immune responsive gene 1 (IRG1) and its main effects on cellular metabolism have been attributed to the inhibition of succinate dehydrogenase. Recent studies provided an insight on the mechanism of its activity, showing that itaconate is required for the activation of anti-inflammatory transcription factor NRF2 by LPS in mouse and human macrophages [28].

Indeed, LPS-activated macrophages lacking endogenous itaconate (IRG1KO) show augmented inflammatory responses when compared to LPS activated wild-type macrophages [161]. Moreover, itaconate modulates immune responses through an NRF2-independent mechanism as well. Indeed, through its immunomodulatory and anti-inflammatory properties, DMI reduces peripheral Th1/Th17 differentiation and CNS infiltration and exerts a protective effect in the EAE mouse model of MS. Moreover, on primary microglia, DMI lowers LPS-induced activation and inflammatory cytokine production [162].

An alternative compound that is recently emerging in MS treatment is ethyl pyruvate (EP) [163]. Based on their structures (Figure 4), DMF and EP share similar chemical reactivity by thiol conjugation according to Michael-type addition (Figure 1). EP activates NRF2 in astrocytes as DMF, and inhibits NF-kB–dependent transcription in different cell types, including LPS-stimulated BV2 (microglia) cells and macrophage-like RAW 264.7 cells [164,165,166,167]. Anti-inflammatory and neuroprotective effects of EP have been observed in animal models of sepsis, uveitis, and asthma [168,169,170]. EP has been effective also against different neurological insults, such as ischemia/reperfusion [171], intracerebral hemorrhage-induced brain injury [172], and traumatic brain injury [173]. EP and DMF share a number of MS-relevant anti-inflammatory effects, as they suppress the release of T cell effector cytokines (IFN-γ and IL 17), reduce iNOS expression in LPS stimulated microglia, leading to NF-kB inhibition [174]. Furthermore, like DMF, EP counteracts microglia activation, lowering the number of CD40+ and F4/80+ cells and suppressing phagocytosis [18].

## 7. Conclusions

In our vision of the described multiple mechanisms underlying the DMF action as antioxidant and immunomodulator in neurological diseases, the most promising beneficial effects arise from the alteration of the redox state of microglia cells. Indeed, DMF treatment modifies the functional properties and polarization states of microglia by modulating the GSH redox metabolism and the expression and activation of a cascade of redox enzymes: this strongly induces ferritin uptake, thus promoting the trophic effect of microglia on oligodendrocytes. The therapeutic strategies based on the modulation of microglia functions are promising for the treatment of neurodegenerative diseases, thus the potential use of DMF or similar compounds may aid in reducing neuroinflammation and detrimental glial action. However, further explanation will be necessary to establish the molecular mechanism of action and the effect of DMF on redox activation pathways in microglia. A good strategy in the research of desirable new compounds would be a deeper study on analogue-based drug designed scaffolds, endowed with more favorable binding properties towards hydroxy-carboxylic acid receptors and selective ability in the inhibition of NRF2 pathway.

## Abbreviation List

aconitase 1 (ACO1); Alzheimer’s disease (AD); amyotrophic lateral sclerosis (ALS); blood–brain barrier (BBB); central nervous system (CNS); collapsin response mediator protein 2 (CRMP2); dimethyl fumarate (DMF); dimethyl itaconate (DMI); diroximel fumarate (DRF); ethyl pyruvate (EP); experimental autoimmune encephalomyelitis (EAE); ferritin (Ft); Friedreich’s ataxia (FA); glutaredoxin (GRx); glutathione (GSH); glutathione peroxidase (GPx); glycogen synthase kinase-3β (GSK-3β); heme oxygenasee-1 (HO-1); hereditary hemochromatosis factor (HFE); hydroxycarboxylic acid receptor (HCAR2); immunoresponsive gene 1 (IRG1); inducible nitric oxide synthase (iNOS); iron response proteins (IRP); iron responsive elements (IREs); kelch-like ECH-associated protein (KEAP1); lipopolisaccaride (LPS); magnetic resonance imaging (MRI); monomethyl fumarate (MMF); multiple sclerosis (MS); nuclear factor–κB (NF-κB); nuclear factor (erythroid-derived 2)-like 2 (NRF2); Parkinson’s disease (PD); reactive oxide species (ROS); superoxide dismutase (SOD); superoxide dismutase-2 (SOD2); T-cell immunoglobulin mucin domain 2 protein (TIM-2); transferrin (Tf); transferrin receptor 1 (TfR1); tricarboxylic acid cycle (TCA); untranslated region (UTR); α-synuclein (α-syn).
